# Predicting Continuity of Asthma Care Using a Machine Learning Model: Retrospective Cohort Study

**DOI:** 10.3390/ijerph19031237

**Published:** 2022-01-22

**Authors:** Yao Tong, Beilei Lin, Gang Chen, Zhenxiang Zhang

**Affiliations:** 1 School of Nursing and Health, Zhengzhou University, Zhengzhou 450001, China; yaotong@uw.edu (Y.T.); linda870926@126.com (B.L.); 2 Department of Biomedical Informatics and Medical Education, University of Washington, UW Medicine South Lake Union, 850 Republican Street, Building C, Box 358047, Seattle, WA 98109, USA; 3 Collaborative Innovation Centre for Internet Healthcare, Zhengzhou University, Zhengzhou 450052, China; 4 School of Computer and Artificial Intelligence, Zhengzhou University, Zhengzhou 450001, China

**Keywords:** continuity of care, asthma, predicting, feature engineering, machine learning, retrospective study

## Abstract

Continuity of care (COC) has been shown to possess numerous health benefits for chronic diseases. Specifically, the establishment of its level can facilitate clinical decision-making and enhanced allocation of healthcare resources. However, the use of a generalizable predictive methodology to determine the COC in patients has been underinvestigated. To fill this research gap, this study aimed to develop a machine learning model to predict the future COC of asthma patients and explore the associated factors. We included 31,724 adult outpatients with asthma who received care from the University of Washington Medicine between 2011 and 2018, and examined 138 features to build the machine learning model. Following the 10-fold cross-validations, the proposed model yielded an accuracy of 88.20%, an average area under the receiver operating characteristic curve of 0.96, and an average F1 score of 0.86. Further analysis revealed that the severity of asthma, comorbidities, insurance, and age were highly correlated with the COC of patients with asthma. This study used predictive methods to obtain the COC of patients, and our excellent modeling strategy achieved high performance. After further optimization, the model could facilitate future clinical decisions, hospital management, and improve outcomes.

## 1. Introduction

### 1.1. Background

Continuity of care (COC) is a mode of structured care delivery. It has been shown to offer numerous health benefits for chronic disease management with fewer adverse outcomes [1,2] and reduced costs [3,4]. A patient with a low level of COC involves better ongoing healthcare management. Thus, knowing the level of COC is essential for implementing care interventions. Thus far, its measurements have varied and mostly focused on finding a way to measure the “interpersonal relationship” between patients and collaborators, such as physicians, caregivers, and patients themselves [5,6,7,8]. Despite the availability of different measurements, appropriately obtaining COCs with a generalizable methodology has been underinvestigated. Notably, the predictive model is an artificial intelligence method that can be deployed in the clinic to facilitate decisions prospectively [9]. Using an effective technique to predict the COC of patients would be a breakthrough. However, the high dependence on multidisciplinary knowledge and massive data collection limits this progression [10]. To precisely identify the degree of COC, we used a machine learning classification model to predict the future level of COC of patients and targeted one of the major chronic diseases, asthma.

Asthma is a common chronic disease that would cause poor outcomes if out of continuous control. In the United States, 7.8% of the people have asthma, causing 1,629,469 emergency department (ED) visits; 178,530 hospitalizations; and over 10.0 million deaths annually [11]. Unlike other chronic diseases, it affects a broader age range, indicating that any effective improvement in asthma would benefit more patients. In addition, younger patients with asthma have a lower COC and more often experience episodic exacerbations [12]. By knowing the COC beforehand and improving it using practical methods, as many as 60–75% of the future ED visits and 25% of the hospitalization by patients with asthma can be avoided [13,14,15,16].

### 1.2. Current Research Gap

Previous studies have focused on finding an association between the COC and outcomes in patients with asthma. However, as demonstrated by the literature [17], proper gauging of COC and outcomes should be prioritized before exploring the relationship between them. As identifying the outcomes of patients is much easier than ascertaining the COC, many studies have developed a predictive model for the former [18,19,20,21,22,23,24,25,26,27,28,29,30,31,32,33,34], while limited research has been performed for the latter.

Most importantly, thus far, assessing the COC of patients has relied on historical data [5,6,7,8]. Studies have extracted patients having longitudinal visiting records for several years to measure the COC, such as the continuity of care index (COCI). The existing quantitative methods all used historical data and had a limited sample pool. Moreover, it cannot be obtained for a new patient who has never been in a specific healthcare system. In the University of Washington Medicine (UWM), approximately 40% of the new patients with asthma receive medical care per year, as shown in Table 1. Assuming that using the past COC could predict its future level, the highest prediction accuracy would be less than 60%. Furthermore, the COC of patients is likely to change with time; thus, prior COC cannot represent the future ones for existing patients with 100% accuracy. We calculated the prediction accuracy using the historical COC for outpatients with asthma who received care from the UWM for 5 years. The highest accuracy was 57.94%, as shown in Table 1. Thus, in this study, the baseline prediction accuracy was set to 57.94%. Although this method seemed uncomplicated, it was insufficient. Furthermore, additional barriers would further affect this approach:

1. Clinical research has mostly chosen claims data, electronic health records (EHR), patient surveys, and consultation to collect data. The claims data and the EHRs usually contain complete historical data, however, extracting and cleaning this massive raw data for clinical researchers is complicated. Thus, patient surveys and consultations are more preferable [7,35,36,37,38,39]. However, general consultation data collection is practiced using computers, including telephone calls or emails, depending on the computer system or the operation person. It could be misleading if various researchers shared the same data or if the computer system changed. Although patient surveys could avoid such misrepresentative findings, the small sample size would limit the study. Therefore, the simple method that directly uses the previous COC to represent the future is limited by specific research, and it is not generalized.
2. Several studies have shown that patient demographics and comorbidities are associated with the COC of patients [37,38]. Using these attributes could certainly facilitate the evaluation of COC in some new patients. Current studies mainly focus on investigating the probability that these characteristics would affect the COC; however, they do not implement them accurately to indicate its specific level. In this research, models were developed to explore the feasibility of using demographic and comorbidity attributes to assess the COC.

Notably, a critical intervention for patients after an asthma attack is to invest in care management. It costs over $5000 per person annually [40] and generally enrolls only below 3% of the patients due to resource limitations [41]. The COC is a part of care management. Undeniably, earlier intervention for patients with low COC would achieve better quality and cost-effectiveness of care management. Thus, it is worthwhile to investigate a generalizable predictive methodology to determine the future COC.

### 1.3. Objective

This study was designed to fill the aforementioned research gap. We proposed a machine learning model to predict the future COC for outpatients with asthma. Our final model integrated the EHRs and the administrative data to estimate three possible categorical COC levels: high, moderate, and low.

## 2. Materials and Methods

### 2.1. Data Source

This retrospective cohort study used the EHRs and the administrative data extracted from the UWM, the most extensive academic healthcare system in the State of Washington. The data warehouse has been collecting complete adults’ data from 12 clinics and 3 hospitals since 2011. This study’s patient population included all outpatient visits from 2011 to 2018.

### 2.2. Data Collection and Patient Cohort

**Figure 1:**
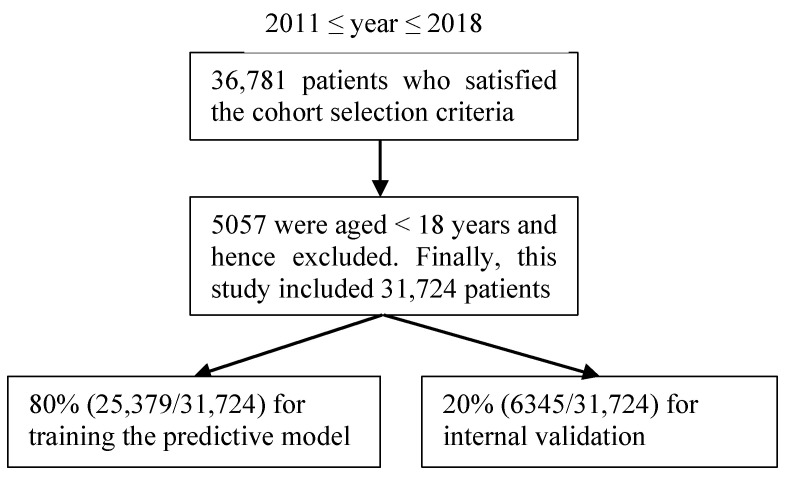
The patient cohort in the study.

The enterprise data warehouse of the UWM contains the original and uncleaned EHRs and administrative data. To ensure data validity, we implemented a data collection and cleaning process before building the predictive model. We identified patients with asthma in a specific year using a minimum of one diagnosis code of asthma in that year: the International Classification of Diseases, Ninth Revision codes 493.9x, 493.8x, 493.1x, and 493.0x; and the International Classification of Diseases, Tenth Revision codes J45.x [20,42,43]. The patient cohort included 31,724 adult outpatients (age ≥ 18 years) with asthma between 1 January 2011 and 31 December 2018; 5057 outpatients (age < 18 years) were excluded. The distribution of this study’s dataset is presented in Figure 1.

### 2.3. Prediction Target

The prediction target in this study was the class of COC score, which represent the level of COC of the patient. To calculate the score of the patients’ COC, the most common COC measurement algorithm, the continuity of care index (COCI) [44], was chosen and divided into three dimensions following the classification strategy of the study [45]. The COCI is composed of the number of visits to each physician and that of distinct physicians consulted [44]. The following general equation represents the COCI of the outpatient visits.

(1) $$\mathrm{COCI}=\sum_{j=1}^{M}n_{j}^{2}/N\left(N-1\right),$$

where *N* refers to the total number of visits to the physicians, *n_j_* denotes the number of visits to physician *j*, and *M* refers to the total number of different physicians.

The COCI ranges from 0 to 1, with a higher score indicating a higher level of COC. In this study, the COCI was classified into three levels: high (0.34–1.00), moderate (0.17–0.33), and low (0.00–0.16). For building an enhanced model, we assigned the numbers 3, 2, and 1 to high, moderate, and low levels, respectively, to represent these three dimensions.

### 2.4. Preprocessing Feature Values

The quality of data and features determines the performance and reliability of a machine learning model; specifically, preprocessing features are essential before training data. In this study, a total of 138 features were examined, describing a large variety of characteristics. Table A1 in the Appendix A describes the details of these features. Except for the demographic features (such as age, gender, race, and ethnicity), those related to medication, insurance, comorbidity, family location, and types of visits were included in this study. Typically, we utilized standardization to process complicated features. We adopted a uniform quantity standard to calculate the structured attributes. An instance of medication features for improved understanding is as follows: a patient who was prescribed medications twice in a specific year. Medications A and B were prescribed for the first time, and A and C for the second time; the total number of prescribed medications was four, and the number of distinct prescribed medications is three this year. In addition, binarization was introduced to quantify broad domain features, including those associated with the family location of patients. Our prior study [46] showed that the 5-mile radius from the patient’s home to the UMW was the threshold distance for the patients who mostly tended to receive care from it. Therefore, we divided the value for this attribute as 1 or 0 to distinguish whether the distance was less than 5 miles.

Every input data instance in the predictive model was independent of the outcome. Therefore, the features corresponding to the number of visits to the physicians were not considered, such as “number of outpatient visits to the patient’s primary care providers”, “number of differing providers the patient saw in outpatient visits,” and “number of differing primary care providers of the patient”. In addition, if some features described similar items, they were integrated into one category. For instance, “primary asthma diagnosis” and “priority asthma diagnosis” were categorized as one entity under “primary asthma diagnosis”.

### 2.5. Modeling

#### 2.5.1. Data Preparation

Most classification algorithms accept only numerical features. Thus, we applied one-hot encoding to transform the categorical features into the numerical ones before they were added to the classifiers. Furthermore, as the COCI is a longitudinal prediction target, the corresponding values were initiated into computing because the patient was first shown in this UWM dataset. The entire 9-year period of this study was from January 2011 to December 2018.

#### 2.5.2. Performance Metrics

For a multiclass classification problem, the prediction accuracy and the area under the receiver operating characteristic curve (AUROC) are two important metrics for evaluating the performance of a predictive model; however, it is not the sole measure to select a proper classifier. We further chose three additional standard metrics: precision, recall, and F1 score for a more precise evaluation. Precision refers to the percentage of positive cases from total predicted cases, recall refers to the percentage of how many total positive cases were predicted correctly with the built model, and F1 score refers to the combined result of precision and recall. The equations for the metrics are as follows:

TP_*i* =_
*T*_*i*_*P*_*i*_, (2)

(3) $${\mathrm{FP}}_{i}=\sum_{j=1,\;j\neq i}^{n}F_{j}P_{i},$$

(4) $${\mathrm{FN}}_{i}=\sum_{j=1,\;j\neq i}^{n}F_{i}P_{j},$$

(5) $$\mathrm{Accuracy}=\sum_{i=1}^{n}TP_{i}/\sum_{i=1}^{n}(TP_{i}+FP_{i}),$$

P*_i_* = *TP_i_*/(*TP_i_* + *FP_i_*), (6)

R*_i_* = *TP_i_*/(*TP_i_* + *FN_i_*), (7)

F1*_i_* = 2*P_i_R_i_*/(*P_i_* + *R_i_*), (8)

Here, P*_i_* refers to precision for class *i*, R*_i_* denotes recall for class *i*, F1*_i_* refers to F1 score for class *i*, TP*_i_* denotes true-positive classifications for class *i,* and FP*_i_* refers to false-positive classifications for class *i*. FN*_i_* refers to false-negative classifications for class *i*. The confusion matrix for multi-class classification is presented in Table 2.

#### 2.5.3. Classification Algorithms

Machine learning classification algorithms predict the probability of an objective variable by inputting labeled data for supervised learning. Our prediction target, the COCI of patients with asthma, was divided into three groups: high (3), moderate (2), and low (1). The machine learning classifiers are the best choice for handling this multiclass classification problem. In order to build a predictive model, this study proposed the use of the extreme gradient boosting (XGBoost) algorithm [47], an efficient and distributed realization of gradient boosting. Typically, the top six classification algorithms are employed to develop the advanced predictive models recognized in the data mining and machine learning literature [47,48]: random forest, *k*-nearest neighbor (*k*-NN), support vector machine (SVM), C4.5 decision tree, XGBoost, and Naive Bayes. Specifically, tree-based algorithms (e.g., random forest, C4.5, and XGBoost) and the SVM are both high-performance tools for classification. The former divides the input space into hyper-rectangles according to the target. The latter uses the kernel trick to convert a linearly nonseparable problem into a linearly separable one, thus prolonging the training duration. The six preliminary algorithms were tested and the XGBoost was selected owing to its superior performance.

The study sample was divided into 80% and 20% for training and internal validation, respectively. We fit them with the six algorithms and applied the 10-fold cross-validations to find the best parameters. The parameters tuned in the experiments for each model are as follows: the balanced or not of class weight, the number of trees, and split criterion measure in random forest; the number of neighbors in the *k*-NN; the balanced or not of class weight, regularization strength, and kernel function in the SVM; the class weight and trees’ maximum depth in the C4.5 and the XGBoost; and the prior probabilities and likelihoods of different classes in the Naive Bayes. The other parameters were automatically set by each algorithm.

#### 2.5.4. Evaluating the Superiority of the Final Model

Overall, 138 features were used to build the final model. Checking more types of features was undoubtedly an essential part of the modeling strategy. As this study was innovative, it was necessary to investigate whether an uncomplicated use of patients’ demographic or comorbidity features to predict the future COC would also be effective. We constructed two additional models using the same patient cohort, prediction target, feature preprocessing method, and machine learning algorithm. The difference between these two models and the final one was the number of features. We named “model_2” as the second model using only demographic features, and “model_3” as the third one using demographic and comorbidity features. The details of the features are listed in Table A2 and Table A3 of the Appendix A.

The purpose of these two models was to examine whether the final model was superior to the simpler models. It was unnecessary to use as many comorbidity features as the final model when the model_3 was built. Furthermore, most clinical studies could not obtain complete comorbidity information by patient surveys or consultations. Thus, we chose 10 asthma-related comorbidity features to build model_3.

## 3. Results

### 3.1. Distributions of the COCI and the Data Instances

Table 3 presents the distributions of the COCI classes and the data instances. During the entire study period, 40.68% (12,905/31,724), 5.69% (1804/31,724), and 53.63% (17,015/31,724) of the data instances indicated low (COCI class = 1), moderate (COCI class = 2), and high COC levels (COCI class = 3), respectively.

### 3.2. Characteristics of the Patient Cohort

Table 4 shows the characteristics of the patient cohort. We computed the *p*-value using the chi-square test [42] to evaluate the statistical differences of the data instances. As displayed in Table 4, most characteristics of the patients presented statistically significantly different distributions (*p* < 0.001) among the three COCI classes, with the exception of the occurrence of bronchopulmonary dysplasia (*p* = 0.99) and cystic fibrosis (*p* = 0.02) in the patient.

### 3.3. Classification Results

#### 3.3.1. Performance Results of Various Machine Learning Models

In this study, the dataset was randomly divided into 80% and 20% as a training and test set, respectively. For comparison purposes, five additional models with the random forest, *k*-NN, SVM, C4.5, and Naive Bayes were evaluated using 10-fold cross-validations under the same sample of the training and test sets. The average values of accuracy, precision, recall, F1 score, and the AUROC of the six models are listed in Table 5. The baseline accuracy calculated using the direct method mentioned previously is listed in Table 5 for an improved comparison.

For a multiclass classification problem, high accuracy and the AUROC guarantee good performance for a predictive model. Furthermore, recall and precision are able to indicate critical factors for imbalanced datasets. Higher recall and precision indicate that additional instances were identified correctly. Notably, the F1 score is the weighted average of recall and precision. Thus, we considered the F1 score, accuracy, and the AUROC as assessments of the prediction performance.

**Figure 2:**
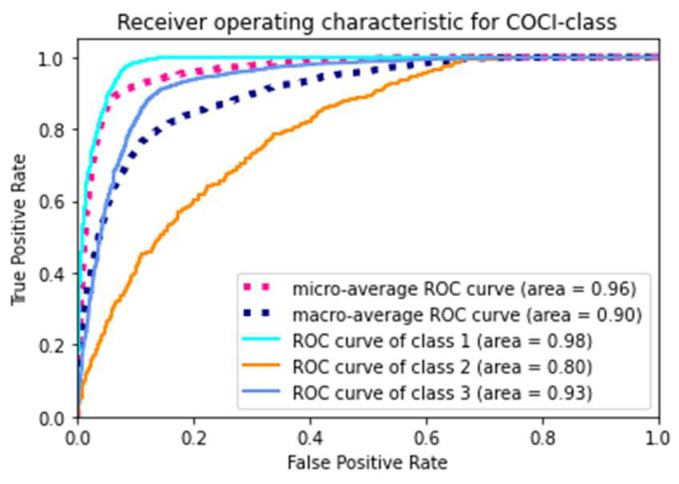
The receiver operating characteristic curve of the final model.

**Figure 3:**
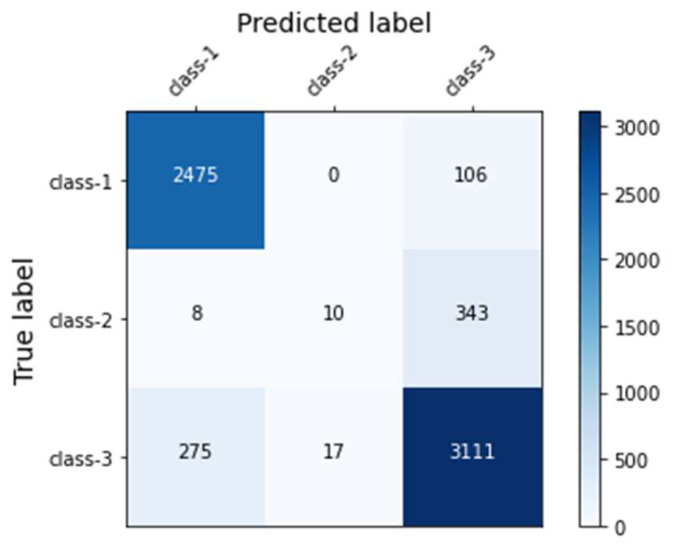
The confusion matrix of the final model.

Across the models, our final model using the XGBoost classifier yielded the highest accuracy (88.20%), the highest F1 score (0.86), and the highest AUROC (0.96). Figure 2 presents the ROC curves of the model. The model gained a microaverage AUROC of 0.96 and a macroaverage AUROC of 0.90, respectively. Specifically, the AUROC of each class of the final model yielded 0.98, 0.80, and 0.93 for classes 1, 2, and 3, respectively. The confusion matrix of the final model is presented in Figure 3. In addition, we tuned a total of 138 features into the XGBoost classifier that was able to automatically compute each feature’s importance value based on its allocated contribution to the model [49]. Our final model was built with 127 features selected by the XGBoost, as listed in Table A4 of the Appendix A, in descending order of the importance values. The XGBoost automatically filtered noncontributing features.

#### 3.3.2. Superiority Evaluation Results

**Figure 4:**
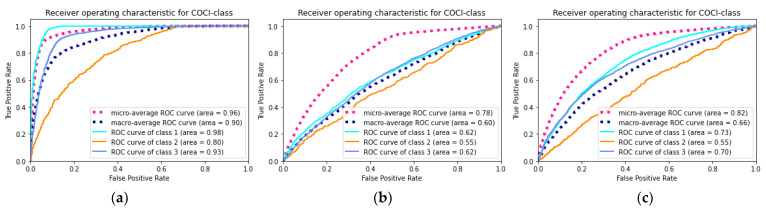
The receiver operating characteristic curve of the models. (**a**) The final model built by full features; (**b**) the model_2 built by only demographic features; (**c**) the model_3 built by demographic and comorbidity features.

The superiority evaluation study examined the performance of two simpler models built with fewer features. With the exception of the features, the dataset, prediction target, and modeling strategy were all consistent with the final model. The comparison results are presented in Table 6 and Figure 4. The final model yielded the best performance among all the metrics.

## 4. Discussion

### 4.1. Principal Findings

In this study, a machine-learning model was developed to predict the future COC of patients with asthma. For enhanced identification and to calculate its level, the COCI was selected, which is the most common algorithm employed by patients and physicians. The XGBoost model yielded the best performance, including the highest accuracy, AUROC, and F1 score. XGBoost won in this study because of its superior big data processing capability. Nevertheless, other algorithms, such as random forest, performed appropriately as well based on the UWM data, owing to our excellent modeling strategy of feature engineering. Furthermore, solely using demographic or comorbidity features to assess the COC was inadequate, further validating the superiority of the modeling strategy. Generally, this study fills the research gap on the use of the predictive method to obtain the COC of patients with asthma that could facilitate the clinical decision-making and allocation of resources, eventually improving patient outcomes.

Overall, 138 features were assessed, and 92.03% (127/138) were used in the final model. Notably, most of the top 30 features in Table A4 of the Appendix A were related to the severity of asthma, comorbidities, insurance, and age, precisely consistent with prior research on the factors associated with care continuity [50].

### 4.2. Comparison with Prior Work

This study fills the research gap of predictive model construction for estimating patients’ COCs; thus, prior works relevant to it are limited. Nevertheless, the use of machine learning to improve patient outcomes, such as disease or poor outcome prediction, has been studied broadly to date. In the research on predicting future outcomes of patients with asthma [18,19,20,21,22,23,24,25,26,27,28,29,30,31,32,33,34], the AUROC ranged from 0.70 to 0.90. The highest AUROC with 0.98 for the low COC level patients in this study obtained better performance than the other models. When building a clinical machine learning model, a similar modeling strategy is usually chosen; although the prediction targets are not compatible, the extensive and effective features and the massive data fed into the model facilitate yielding a higher AUROC. Moreover, our precise data extraction strategy for identifying the prediction target contributes to excellent performance.

Notably, different models were built on varying patient cohorts and predicting similar targets. Some studies have used data from patient surveys and self-report outcomes to analyze the COC of those with asthma. This study employed the EHRs and the administrative data that contained greater clinical characteristics for enhanced profiling. The final predictive model was built using the XGBoost, a state-of-the-art machine-learning algorithm. Compared with statistical approaches (linear model) such as logistic regression, the XGBoost (ensemble model) can intensify the prediction performance with less fundamental assumptions on data distribution [51,52]. As partial evidence for this, we built two additional simpler models to validate our modeling strategy’s superiority and generalizability. The excellent performance demonstrated the feasibility of using our final model to predict the COC of patients with asthma.

### 4.3. Clinical Significance and Potential Use

Our model showed excellent performance in predicting the level of COC for patients with asthma. After working with the healthcare system’s Information Technology (IT) team, we can deploy the model by publishing it as a web service, and the model would benefit both patients and hospital management. Knowing the level of future COC could facilitate the design of an improved objective intervention for patients with asthma. In addition, investing in patients with high COC and providing long-term health services has practically been the goal of all policymakers and healthcare organizations to save colossal costs.

Furthermore, once the patients were identified as having low COC, interventions could have been implemented to prevent it. In the clinical environment, interventions such as adding the COC score to the medical record, investing the patient into care management, and increasing the frequency of follow-up should be considered. Moreover, research has found that adjusting insurance policies, roles, and care delivery strategies can improve the COC [53]. However, such interventions for continuity of asthma care are multifaceted, as they [54] consist of several components such as: (1) interdisciplinary cooperation including interdisciplinary care standards, case conferences, and shared patient management tools; (2) the education of patients and their caregivers and the decision-making involved; (3) implementation of measurable goals of a care plan; (4) allocation of supplemental resources; and (5) coordination of care in the transition. These various components must be considered before designing the interventions.

Literature [55,56] has demonstrated that reimbursement and copayments are associated with improving COC; thus, if this insurance policy is reasonable, such as offering higher reimbursement or lower copayments, both patients and physicians could benefit.

Care delivery strategies can be flexible because thus far, no standard has been used uniformly, and numerous factors should be considered. Investing patients in case management is an effective strategy for improving the COC [57]. Typically, case management is a client-faced approach for promoting cooperation among services, benefits, and opportunities. Activities are designed by case managers to optimize the functioning of people with multiple needs [57]. Regarding asthma care, nurses could be case managers who devote themselves to improving the COC of patients [58]. The literature [59] has indicated that scheduling nurse-led follow-up care appointments increases the COC. A study [60] that recruited 1000 patients (including those with asthma) found that making earlier follow-up care appointments (after discharge is the best time) improved the attendance of the appointment. Similar results were found in research [61] that provided the patients with asthma a free 5-day medication tutorial such as prednisone, a 2-day telephone reminder for making an appointment, and travel vouchers for revisiting their providers that would significantly increase the COC.

Nevertheless, the follow-up care appointments made by providers obtained improved adherence compared to scheduling by patients themselves. The literature [62] has shown that 29% of the cases did not revisit when the care facility stopped the follow-up appointments. Therefore, education is necessary for patients and their caregivers. Numerous studies [58,63,64,65,66] have shown that education programs in various forms, such as home-, web-, and telephone-based, have positive influences on asthma control. These programs increase the conjunction between patients or their caregivers and healthcare facilities [67]. Despite no evidence proving whether education is directly associated with the COC, the conjunction increased by the education programs supports this viewpoint, as the COC is essentially a mode of care delivery coordinated by patients and healthcare facilities. Thus, future research could investigate the association between the COC and asthma education.

### 4.4. Limitations

This study has several limitations that could be potential topics for future research:

1. This study chose the COCI, an algorithm that mainly focuses on the relationship between patients and physicians to assess the COC of patients. In the future, it is possible to evaluate the COC using other methods by considering the interpersonal, geographical, socioeconomic, educational, and cultural aspects;
2. The UWM is an academic healthcare system located in an urban area, and we could not access the data outside it. Thus, this study method’s generalizability to other healthcare systems and rural areas could be further examined;
3. This study’s model was built using a machine learning algorithm, which is a black-box approach, without any explanation. In the future, implementing a rule-based method to explain the predictions would benefit clinical use.

## 5. Conclusions

This study fills the research gap in building a predictive model on massive and longitudinal data to estimate the patients’ COCs. The excellent modeling strategy of assessing many features and precise prediction target identification obtained a high performance. This methodology has the potential to be generalized and benefit more diseases. After further optimization, the model could facilitate future clinical decisions, hospital management, and improve outcomes.

## Figures and Tables

**Table 1:** The distributions of the patients and the data instances between 2014 to 2018.

The Category of Outpatient with Asthma Who Received Care from UWM ^1^	Number in 2014 (N = 9635), *n* (%)	Number in 2015 (N = 10,192), *n* (%)	Number in 2016 (N = 11,017), *n* (%)	Number in 2017 (N = 12,151), *n* (%)	Number in 2018 (N = 12,894), *n* (%)
Returned patients	4926 (51.13)	6010 (58.97)	6453 (58.57)	7549 (62.13)	8186 (63.49)
New patients	4709 (48.87)	4182 (41.03)	4564 (41.43)	4602 (37.87)	4708 (36.51)
Same COCI ^2^ (accuracy)	4708 (48.86)	5667 (55.60)	6041 (54.83)	6965 (57.32)	7471 (57.94)

^1^ UWM: university of Washington medicine. ^2^ COCI: continuity of care index.

**Table 2:** The confusion matrix.

Prediction Class	Predicted COCI-Class = 1 ^2^	Predicted COCI-Class = 2 ^3^	Predicted COCI-Class = 3 ^4^
COCI ^1^-class = 1	*T*_1_ *P*_1_	*F*_1_ *P*_2_	*F*_1_ *P*_3_
COCI-class = 2	*F*_2_ *P*_1_	*T*_2_ *P*_2_	*F*_2_ *P*_3_
COCI-class = 3	*F*_3_ *P*_1_	*F*_3_ *P*_2_	*T*_3_ *P*_3_

^1^ COCI: the continuity of care index. ^2^ class = 1: the low level of COCI. ^3^ class = 2: the moderate level of COCI. ^4^ class = 3: the high level of COCI.

**Table 3:** The distributions of the COCI category and the data instances between 2011 to 2018.

Data Category	Data Instances Connecting to Asthma COCI (N = 31,724), *n* (%)
Number of class = 1	12,905 (40.68%)
Number of class = 2	1804 (5.69%)
Number of class = 3	17,015 (53.63%)

**Table 4:** The characteristics of the patients at the UWM from 2011 to 2018.

Characteristics of Patients	Data Instances (*N* = 31,724), *n* (%)	Data Instances Connecting to Asthma COCI Class = 1 (*N* = 12,905), *n* (%)	Data Instances Connecting to Asthma COCI Class = 2 (*N* = 1804), *n* (%)	Data Instances Connecting to Asthma COCI Class = 3 (*N* = 17,015), *n* (%)	*p*-Value
**Age**					
<40	11,611 (36.60)	5738 (44.46)	759 (42.07)	5114 (30.06)	<0.001
40 to 65	14,839 (46.78)	5524 (42.81)	834 (46.23)	8481 (49.84)	
65+	5274 (16.62)	1643 (12.73)	211 (11.70)	3420 (20.10)	
**Gender**					
Male	11,200 (35.30)	4720 (36.57)	643 (35.64)	5837 (34.31)	<0.001
Female	20,521 (64.69)	8182 (63.40)	1161 (64.36)	11,178 (65.69)	
Unknown or not reported	3 (0.01)	3(0.02)	0 (0.00)	0 (0.00)	
**Race**					
American Indian or Alaska native	500 (1.58)	174 (1.35)	28 (1.55)	298 (1.75)	<0.001
Asian	2909 (9.17)	1150 (8.91)	174 (9.65)	158 (0.93)	
Black or African American	2911 (9.18)	890 (6.90)	230 (12.75)	1791 (10.53)	
Native Hawaiian or other Pacific islander	302 (0.95)	114 (0.88)	24 (1.33)	164 (0.96)	
Other	82 (0.26)	49 (0.38)	3 (0.17)	30 (0.18)	
White	22,361 (70.49)	9058 (70.19)	1232 (68.29)	12,071 (70.94)	
Unknown or not reported	2659 (8.38)	1470 (11.39)	113 (6.26)	1076 (6.32)	
**Ethnicity**					
Hispanic	1625 (5.12)	614 (4.76)	100 (5.54)	911 (5.35)	<0.001
Non-Hispanic	25,783 (81.27)	9757 (75.61)	1554 (86.14)	14,472 (85.05)	
Unknown or not reported	4316 (13.60)	2534 (19.64)	150 (8.31)	1632 (9.59)	
**Insurance**					
Private	23,446 (73.91)	9224 (71.48)	1374 (76.16)	12,848 (75.51)	<0.001
Public	14,322 (45.15)	4833 (37.45)	893 (49.50)	8596 (50.52)	<0.001
Self-paid or charity	1289 (4.06)	298 (2.31)	109 (6.04)	882 (5.18)	<0.001
**No. of years from the first encounter related to asthma in the data set**					
≤3	25,527 (80.47)	12,901 (99.97)	1179 (65.35)	11,447 (67.28)	<0.001
>3	6197 (19.53)	4 (0.03)	625 (34.65)	5568 (32.72)	
**Asthma medication prescription**					
Inhaled corticosteroid	19,734 (62.21)	5482 (42.48)	1259 (69.79)	12,993 (76.36)	<0.001
Inhaled corticosteroid/long-acting beta-2 agonist combination	16,537 (52.13)	4261 (33.02)	1080 (59.87)	11,196 (65.80)	<0.001
Leukotriene modifier	6784 (21.38)	1463 (11.34)	429 (23.78)	4892 (28.75)	<0.001
Long-acting beta-2 agonist	8502 (26.80)	1881 (14.58)	548 (30.38)	6073 (35.69)	<0.001
Mast cell stabilizer	51 (0.16)	13 (0.10)	3 (0.17)	35 (0.21)	<0.001
Short-acting inhaled beta-2 agonist	29,019 (91.47)	11,009 (85.31)	1770 (98.12)	16,240 (95.45)	<0.001
Systemic corticosteroid	15,556 (49.04)	4491 (34.80)	950 (52.66)	10,115 (59.45)	<0.001
**Comorbidity**					
Allergic rhinitis	8421 (54.13)	1872 (14.51)	602 (33.37)	5947 (34.95)	<0.001
Anxiety or depression	10,891 (34.33)	3008 (23.31)	758 (42.02)	7125 (41.87)	<0.001
Bronchopulmonary dysplasia	3 (0.01)	1 (0.01)	1 (0.06)	1 (0.01)	0.99
Chronic obstructive pulmonary disease	2265 (7.14)	471 (3.65)	143 (7.93)	1651 (9.70)	<0.001
Cystic fibrosis	36 (0.11)	16 (0.12)	4 (0.22)	16 (0.09)	0.02
Eczema	3138 (9.89)	606 (4.70)	223 (12.36)	2309 (13.57)	<0.001
Gastroesophageal reflux	6571 (20.71)	1408 (10.91)	408 (22.62)	4755 (27.95)	<0.001
Obesity	3962 (12.49)	829 (6.42)	285 (15.80)	2848 (16.74)	<0.001
Sinusitis	5906 (18.62)	1392 (10.79)	357 (19.79)	4157 (24.43)	<0.001
Sleep apnea	3192 (10.06)	623 (4.83)	208 (11.53)	2361 (13.88)	<0.001

**Table 5:** Prediction performance of various machine learning models.

Model	Accuracy	Precision	Recall	F1 Score	AUROC
Baseline	57.94%	-	-	-	-
C4.5	87.37%	0.84	0.87	0.85	0.90
*k*-NN	59.62%	0.60	0.60	0.60	0.63
Naive Bayes	46.04%	0.71	0.46	0.38	0.88
SVM	84.90%	0.81	0.85	0.82	0.87
Random forest	87.87%	0.86	0.87	0.85	0.94
XGBoost (our final model)	88.20%	0.85	0.88	0.86	0.96

**Table 6:** Performance results between the final and compared models.

Model	Accuracy	Precision	Recall	F1 Score	AUROC
Baseline	57.94%	-	-	-	-
Model_2	57.42%	0.54	0.57	0.54	0.78
Model_3	63.75%	0.60	0.64	0.62	0.82
Final model	88.20%	0.85	0.88	0.86	0.96

## Appendix A

**Table A1:** The list of candidate features considered in the final model.

Feature Category	Features
Features on patient demographics	Race; age; ethnicity (Hispanic/non-Hispanic); marital status (married, divorced, separated, single, widowed, or partnered); gender; and language.
Features that are concerning diagnoses and calculated based on ICD-10 and ICD-9 diagnosis codes	No. of diagnoses of asthma; No. of diagnosis codes concerns ICD-10 and ICD-9; no. of primary asthma diagnoses; no. of years since first diagnosed with asthma in the data set; no. of diagnoses of status asthmaticus; whether the latest diagnoses of asthma is a primary one; the severity of the latest asthma diagnoses; the utmost exacerbation severity among all of the asthma diagnoses; no. of diagnoses of acute asthma; no. of days since the latest asthma diagnosis; the severity of the utmost severity of asthma diagnosis; no. of diagnosis codes of noncompliance with the medication regimen; no. of days since the latest diagnoses with acute asthma or status asthmaticus; the latest diagnoses of asthma that indicate the exacerbation severity (uncomplicated, exacerbation, or asthmaticus); allergic rhinitis; sleep apnea; gastrostomy tube; immunoglobulin A (IgA) deficiency; cystic fibrosis; cirrhosis; chronic obstructive pulmonary disease (COPD); no. of years since first diagnosed with COPD in the data set; vitamin D deficiency; upper respiratory tract infection; congestive heart failure; esophagitis; anxiety or depression; ischemic heart disease; eczema; obesity; paraplegia or hemiplegia; decreased tone; metastatic solid tumor; increased tone; pneumonia; vocal cord dysfunction; psoriasis; anaphylaxis; vasculitis; gastrointestinal obstruction; inflammatory bowel disease; dementia; mental disorder; breathing abnormality like dyspnea; mild liver disease; Alzheimer’s or Parkinson’s disease; pregnancy; myocardial infarction; folate deficiency; gastrointestinal bleeding; malignancy; moderate or severe liver disease; peripheral vascular disease; acquired immunodeficiency syndrome; peptic ulcer disease; cerebrovascular disease; gastroesophageal reflux; substance use; rheumatic disease; renal disease; diabetes without chronic complication; cataract; bronchopulmonary dysplasia; tracheostomy; sinusitis; and family history of asthma.
Features concerning medications	The sum of medications ordered; the sum of various medications ordered; no. of medication orders; the sum of medication refills authorized; the sum of asthma medications ordered; the sum of units of medications ordered; no. of medication orders concerning asthma; the sum of asthma medication refills authorized; the sum of various asthma medications ordered; the sum of units of medications ordered concerning asthma; no. of medication prescribers; no. of medication prescribers concerning asthma; the sum of short-acting beta-2 agonists (SABAs) ordered; the sum of units of SABAs ordered; the sum of refills authorized for SABAs; the sum of systemic corticosteroids ordered; the sum of units of systemic corticosteroids ordered; the sum of refills authorized for systemic corticosteroids; no. of reliever orders concerning asthma; the sum of refills authorized for asthma relievers; the sum of relievers ordered concerning asthma; the sum of diverse asthma relievers ordered; the sum of units of relievers ordered concerning asthma that are neither SABAs nor systemic corticosteroids; the sum of units of relievers ordered concerning asthma; the sum of relievers ordered concerning asthma that are neither SABAs nor systemic corticosteroids; the sum of controllers ordered concerning asthma; no. of controller orders concerning asthma; the sum of various asthma controllers ordered; the sum of units of controllers ordered concerning asthma; the sum of refills authorized for asthma controllers; the sum of refills authorized for inhaled corticosteroids; the sum of inhaled corticosteroids ordered; the sum of units of inhaled corticosteroids ordered; the sum of refills authorized for mast cell stabilizers; the sum of ordered for mast cell stabilizers; the sum of units of ordered for mast cell stabilizers; the sum of nebulizer medications ordered; no. of nebulizer medication orders; the sum of various nebulizer medications ordered; the sum of units of ordered concerning nebulizer medications; the sum of refills authorized for nebulizer medications; whether spacer was used; and whether nebulizer was used.
Features concerning insurances	Whether the patient enrolled in any public insurance; whether the patient was paid by charity or self-paid; and whether the patient enrolled in any private insurance. We calculate the features related to insurances on the last day of the specific year.
Features concerning the visit types of the patient	No. of ED visits; the latest length of stay of ED visit; no. of ED visits concerning asthma; the average ED visit’s length of stay; no. of outpatient visits; no. of all type (ED visit, hospital stay, and outpatient visit) of visits; no. of outpatient visits who diagnosed with asthma as the primary diagnosis; the total length of hospital stay; no. of hospitalizations; the average a hospitalization’s length of stay; the latest visit’s admission type (trauma, urgent, elective, or emergency); the most emergent hospital admission type among all of the visits; no. of prime asthma visits; and the latest visit’s type (ED visit, hospital stay, or outpatient visit). According to our prior paper [34], we defined a prime asthma visit as an ED visit with a diagnosis of asthma, a hospitalization with a diagnosis of asthma, or an outpatient visit with a primary diagnosis of asthma. An outpatient visit with only a secondary diagnosis of asthma was assigned as a minor asthma visit.
Features concerning appointment and visit status	No. of no shows; and no. of canceled appointments.
Features concerning the family location of the patient	Whether the distance from the patient’s home to UMW is less than 5-miles.

**Table A2:** The list of candidate features considered in model_2.

Feature Category	Features
Features on patient demographics	Race; age; ethnicity (Hispanic/non-Hispanic); marital status (married, divorced, separated, single, widowed, or partnered); gender; and language.

**Table A3:** The list of candidate features considered in model_3.

Feature Category	Features
Features on patient demographics	Race; age; ethnicity (Hispanic/non-Hispanic); marital status (married, divorced, separated, single, widowed, or partnered); gender; and language.
Features that are concerning diagnoses and calculated based on ICD-10 and ICD-9 diagnosis codes (Comorbidity features)	Allergic rhinitis; sleep apnea; cystic fibrosis; COPD; anxiety or depression; eczema; obesity; gastroesophageal reflux; bronchopulmonary dysplasia; and sinusitis.

**Table A4:** The features used in our final model and their importance values.

Rank	Feature	Importance Calculated as the Feature’s Apportioned Contribution to the Model
1	No. of diagnoses	0.5311
2	No. of outpatient visits who diagnosed with asthma as the primary diagnosis	0.0792
3	No. of asthma diagnoses	0.0102
4	Whether the latest diagnosis of asthma is a primary one	0.0078
5	The severity of the latest asthma diagnoses	0.0065
6	No. of prime asthma visits	0.0061
7	No. of medication orders	0.0059
8	The sum of refills authorized for asthma controllers;	0.0057
9	No. of years since first diagnosed with asthma in the data set	0.0057
10	The sum of units of controllers ordered concerning asthma	0.0050
11	The severity of the utmost severity of asthma diagnosis	0.0044
12	Whether the patient has AIDS/HIV	0.0044
13	Whether the patient has mental disorder	0.0043
14	No. of ED visits concerning asthma	0.0041
15	The sum of units of relievers ordered concerning asthma	0.0039
16	Whether the patient has sinusitis	0.0038
17	The sum of units of SABAs ordered	0.0038
18	Whether the patient has substance use	0.0038
19	No. of outpatient visits	0.0038
20	No. of primary asthma diagnoses	0.0038
21	No. of all type (ED visit, hospital stay, and outpatient visit) of visits	0.0038
22	The sum of asthma medication refills authorized	0.0038
23	The sum of refills authorized for SABAs	0.0036
24	The total length of hospital stay	0.0036
25	No. of ED visits	0.0035
26	The sum of units of inhaled corticosteroids ordered	0.0035
27	Age	0.0035
28	Whether the patient is single	0.0035
29	Whether the patient is Hispanic	0.0035
30	The sum of refills authorized for inhaled corticosteroids	0.0035
31	No. of reliever orders concerning asthma	0.0035
32	Whether the patient has rhinitis	0.0035
33	Whether the patient has vitamin D deficiency	0.0035
34	Whether the patient was paid by charity or self-paid	0.0034
35	Whether the patient has psoriasis	0.0034
36	The sum of various asthma medications ordered	0.0034
37	Whether the distance from the patient’s home to UMW is less than 5-mile	0.0034
38	No. of diagnoses of status asthmaticus	0.0034
39	No. of controller orders concerning asthma	0.0034
40	No. of canceled appointments	0.0033
41	Whether the patient has dyspnea	0.0033
42	The sum of diverse asthma relievers ordered	0.0033
43	Whether the patient has pneumonia	0.0032
44	Whether the patient has rheumatic_disease	0.0032
45	No. of medication orders	0.0032
46	The sum of units of medications ordered	0.0032
47	The sum of refills authorized for systemic corticosteroids	0.0032
48	Whether the patient has COPD	0.0032
49	The sum of refills authorized for asthma relievers	0.0032
50	No. of no shows	0.0031
51	The sum of various asthma controllers ordered	0.0031
52	Whether the patient has folate deficiency	0.0031
53	The sum of units of systemic corticosteroids ordered	0.0031
54	The sum of SABAs ordered	0.0031
55	the average a hospitalization’s length of stay	0.0031
56	The sum of various medications ordered	0.0031
57	Whether the patient is pacific islander	0.0031
58	No. of diagnoses of acute asthma	0.0031
59	The sum of units of medications ordered	0.0031
60	No. of medication prescribers	0.0030
61	Whether the patient is married	0.0030
62	No. of medication prescribers concerning asthma	0.0030
63	Whether the patient is separated	0.0030
64	The sum of medication refills authorized	0.0030
65	Whether the patient has sleep apnea	0.0030
66	The sum of various nebulizer medications ordered	0.0030
67	Whether the patient has myocardial infarction	0.0030
68	The average ED visit’s length of stay	0.0030
69	Whether the patient has AP dementia	0.0029
70	Whether the patient has moderate or severe liver disease	0.0029
71	Whether the patient is female	0.0029
72	The utmost exacerbation severity among all of the asthma diagnoses	0.0029
73	Whether the patient is pregnant	0.0029
74	No. of diagnosis codes of noncompliance with the medication regimen	0.0029
75	Whether nebulizer was used	0.0029
76	Whether the patient is White	0.0029
77	Whether the patient has obesity diagnosis code	0.0028
78	Whether the patient enrolled in any public insurance	0.0028
79	No. of nebulizer medication orders	0.0028
80	Whether the patient has ischemic heart disease	0.0028
81	Whether spacer was used	0.0028
82	Whether the patient has peripheral vascular disease	0.0028
83	Whether the patient is widowed	0.0028
84	Whether the patient has inflammatory bowel disease	0.0028
85	Whether the patient enrolled in any private insurance	0.0028
86	Whether the patient has gastrointestinal bleeding	0.0028
87	Whether the patient has renal disease	0.0028
88	Whether the patient is Asian	0.0028
89	Whether the patient has reflux	0.0027
90	Whether the patient is Black	0.0027
91	Whether the patient has esophagitis	0.0027
92	The sum of units of ordered concerning nebulizer medications	0.0027
93	No. of years since first diagnosed with COPD in the data set	0.0027
94	Whether the patient has anxiety depression	0.0027
95	The sum of systemic corticosteroids ordered	0.0027
96	The severity of the utmost severity of asthma diagnosis	0.0027
97	Whether the patient has mild liver disease	0.0026
98	Whether the patient is divorced	0.0026
99	The sum of relievers ordered concerning asthma that are neither SABAs nor systemic corticosteroids	0.0026
100	The sum of units of inhaled corticosteroids ordered	0.0026
101	Whether the patient has vocal cord dysfunction	0.0026
102	Whether the patient speaks Spanish	0.0025
103	Whether the patient has eczema	0.0025
104	Whether the patient has diabetes with chronic complication	0.0025
105	Whether the patient has malignancy	0.0024
106	Whether the patient has gastrostomy tube	0.0023
107	Whether the patient has URTI	0.0023
108	The sum of units of relievers ordered concerning asthma that are neither SABAs nor systemic corticosteroids	0.0023
109	Whether the patient has anaphylaxis	0.0023
110	Whether the patient has metastatic	0.0022
111	Whether the patient has cerebrovascular	0.0022
112	Whether the patient has vasculitis	0.0022
113	No. of hospitalizations	0.0022
114	The sum of refills authorized for nebulizer medications	0.0022
115	Whether the patient has cirrhosis	0.0020
116	Whether the patient has diabetes without chronic complication	0.0020
117	Whether the patient speaks English	0.0020
118	Whether the patient has congestive heart failure	0.0019
119	Whether the patient has decreased tone	0.0018
120	Whether the patient has cystic fibrosis	0.0017
121	Whether the patient has increased tone	0.0014
122	Whether the patient has GI obstruction	0.0012
123	Whether the patient has hemiplegia	0.0012
124	Whether the patient has IgA deficiency	0.0009
125	Whether the patient has peptic ulcer disease	0.0008
126	Whether the patient has Charlson dementia	0.0008
127	Whether the patient has bronchiolitis	0.0005
